# A smartphone based attentive eating intervention for energy intake and weight loss: results from a randomised controlled trial

**DOI:** 10.1186/s12889-019-6923-x

**Published:** 2019-05-21

**Authors:** Victoria Whitelock, Inge Kersbergen, Suzanne Higgs, Paul Aveyard, Jason C. G. Halford, Eric Robinson

**Affiliations:** 1 Department of Psychological Sciences, University of Liverpool, Eleanor Rathbone Building, Bedford Street South, Liverpool, L69 7ZA UK; 2 Cancer Intelligence, Cancer Research UK, Angel Building, 407 St John Street, London, EC1V 4AD UK; 3 School of Health and Related Research, University of Sheffield, 30 Regent Street, Sheffield, S1 4DA UK; 4 The School of Psychology, University of Birmingham, Edgbaston, Birmingham, B15 2TT UK; 5 Nuffield Department of Primary Care Health Services, University of Oxford, Oxford, UK

**Keywords:** Attentive eating, Weight loss, Smartphone application, Ehealth, Mhealth, Food intake, Obesity, Overweight, Focused attention

## Abstract

**Background:**

Laboratory studies suggest that eating more ‘attentively’ (e.g. attending to food being eaten and recalling eating episodes) can reduce food intake among participants with both healthy weight and overweight. The aim of this trial was to assess whether a smartphone application that encourages a more attentive eating style reduces energy intake and promotes weight loss.

**Methods:**

In an open-label, single centre, parallel groups, individually randomised controlled trial, 107 adults with overweight/obesity in Merseyside, UK used an attentive eating smartphone application along with standard dietary advice (intervention group) or standard dietary advice only (control group) for 8 weeks. The primary outcomes were change in body weight at 8 weeks and energy intake at 4 and 8 weeks. Additional outcomes included self-reported eating behaviours measured at 8 weeks. Differences between groups were assessed with linear regression (adjusted) using multiple imputation for missing data. Study protocol registered prospectively at (10.17605/osf.io/btzhw).

**Results:**

There was no significant difference between the intervention and control group in weight lost at 8 weeks, or change in self-reported 24 h or objective taste-test energy intake at 4 or 8 weeks. Mean weight loss in the intervention group (*n* = 53) was 1.2 kg and 1.1 kg in the control group (*n* = 54), adjusted difference of − 0.10 (− 1.6 to 1.3) kg. Self-reported eating behaviours at 8 weeks also did not differ across groups. The intervention was largely used as intended and a per protocol analysis confined to participants in the intervention group that used the attentive eating smartphone application regularly and as intended also showed no effect on energy intake or weight loss.

**Conclusions:**

A smartphone based attentive eating intervention and standard dietary advice did not result in reduced energy intake or greater weight loss at 4 or 8 week follow-up than standard dietary advice alone.

**Trial registration:**

ClinicalTrials.gov, NCT03602001. Registered retrospectively on 26th July 2018.

Prospectively registered on the Open Science Framework on 11th August 2017.

**Electronic supplementary material:**

The online version of this article (10.1186/s12889-019-6923-x) contains supplementary material, which is available to authorized users.

## Background

Across English-speaking high-income countries, obesity rates currently exceed 30% and this is predicted to rise [1]. The health problems often associated with obesity, including type 2 diabetes, cardiovascular disease, hypertension and several types of cancer are substantial [2–4]. Easily implementable interventions that help people reduce their food intake and aid weight loss are therefore needed. Moreover, with approximately 76% of UK adults now owning a smartphone, smartphones may provide a cost effective platform for delivering behavioural interventions [5].

Eating ‘on the go’ and while distracted may be contributing to overeating. Laboratory studies suggest that eating while distracted can increase concurrent food intake, hunger afterwards, and later snacking [6–9] which may occur because failing to attend to food being eaten impairs memory for what has been eaten. In line with this, laboratory studies also indicate that memory for recent eating episodes are factored into subsequent food intake decisions [10–12]. For example, increasing awareness of recent eating episodes has been found to reduce later snacking [13] and paying more attention to food as it is being consumed has been found to reduce later snacking in most [14–16], (but not all [17]) laboratory studies in participants with healthy weight and overweight. These promising results from the laboratory suggest that an intervention approach that promotes a more ‘attentive’ style of eating by encouraging participants to attend to food being eaten and recall eating episodes may be an effective way of reducing food intake and aiding weight loss [18]. Moreover, because such an approach would not rely on conscious and vigilant calorie counting, it may be relatively acceptable to users [19].

To examine the potential of an attentive eating intervention approach, we previously developed and tested initial feasibility of a smartphone based application that encourages users to eat more attentively [20]. The smartphone application encouraged an attentive eating style by requiring users to photograph their meals and attend to food while eating (utilising present moment awareness [21]). They were also required to review what else they had eaten that day before entering their next meal, with an overall aim of encouraging a more attentive eating style [20]. Participants with overweight and obesity used the application in a small scale feasibility trial and reported in qualitative interviews that they found the smartphone application easy to use, increased their awareness of what they had been eating and that the frequency of application usage was acceptable. To date, there has been no examination of whether an attentive eating based intervention is effective in reducing energy intake and promoting weight loss. The aim of this randomised control trial was to test initial proof of concept for effectiveness of an attentive eating smartphone application to reduce energy intake and promote weight loss.

## Methods

### Design and sample

The trial methods and analysis strategy were pre-registered on the open science framework (10.17605/osf.io/btzhw). This study was a single centre, parallel, two arm, individually randomised 8 week controlled trial. Participants were randomly allocated to receive either an attentive eating smartphone application along with a standard dietary advice booklet and the same dietary advice delivered by text message once a week (intervention group), or the dietary advice booklet and weekly text messages only (control group). Assessments were conducted at baseline, 4 and 8 weeks. The inclusion of basic dietary information and weekly text messages repeating this information in both the control and intervention group ensured that, in line with recommendations of best practice [22, 23], our control group were actively trying to lose weight, but the resources they were provided with were minimal and not expected to promote a large amount of weight loss. In addition, this design ensured that both the control and intervention group were required to use their smartphone during the trial.

A 2 kg difference in weight loss between the two groups across 8 weeks was considered meaningful, with a conservative estimate of the standard deviation of weight loss across conditions of 2.5 kg (based on [24]). To detect this difference in weight loss (alpha level = 0.05, 90% power, GPower 3.1) we required a minimum sample size of *N* = 68. However, due to greater participant interest in the trial than anticipated, we recruited above this number during a 7 week baseline data collection period (see pre-registered protocol).

To be eligibile to take part participants had to be classed as overweight or obese based on their BMI (BMI ≥25.0 kg/m^2^) and report that they would like to lose weight by changing their dietary behaviour. Participants were also required to have no history of eating disorders or food allergies (self-reported by participant), be aged 18–65 years, be fluent English speakers, not taking medication that affects appetite, not pregnant, not scheduled for weight loss surgery during the trial, own an Android/Apple smartphone (Android operating system versions 4.4–7.1, Apple operating system iOS 8–10) and not currently on a structured weight loss programme. Participants with diabetes were able to take part, providing they were not using insulin or other diabetes medication that affects appetite.

### Randomisation and blinding

The randomisation sequence was created using the computer programme ‘random allocation software’ [25] and was stratified by baseline body mass index (BMI, 2 strata: 25–32.5 kg/m^2^; > 32.5 kg/m^2^) with a 1:1 allocation, using random block sizes of 2 and 4. A colleague outside the research team generated this sequence and placed the allocations inside sequentially numbered sealed opaque envelopes (ensuring allocation concealment). Participants were not blind to condition allocation, neither were staff delivering the intervention and collecting baseline and follow-up measures.

### Interventions

#### Standard dietary advice booklet

The booklet contained information and tips adapted from British Heart Foundation materials [26] on healthy eating and weight loss (e.g. components of a balanced diet, reducing calories and lower calorie swaps, consuming fruits and vegetables, avoiding foods high in fat and sugar, drinks, shopping and eating out) and brief information about the importance of physical activity. The intervention materials used can be found on the open science framework: 10.17605/osf.io/btzhw.

#### Weekly text messages

Participants also received once weekly tips via text message that related to content from the dietary advice booklet (the full list of text messages is provided in the pre-registered trial protocol on the open science framework: 10.17605/osf.io/btzhw). Text messages were sent at the same time on the same day each week.

#### Attentive eating application

The approach to designing the attentive eating smartphone application was described in detail in the feasibility trial [20]. The attentive eating application was designed to promote attentive eating by encouraging users to photograph food and drink being consumed and then review this information when making dietary decisions throughout the day. Prior to eating/drinking a food or beverage users accessed the camera function of their phone and selected the meal type they wished to record (breakfast, lunch, dinner, snack, drink, other). Alternatively, users could select a photograph that was already stored on their phone or they could write a description of the food. After an adjustable time period, the application sent a notification reminding users to complete questions about their consumption experience once they had finished the meal. After finishing the meal/drink, users accessed the consumption experience questions and the photograph of the recently consumed food/drink was displayed, with information about the meal type consumed. With this image on the screen, users selected drop down answers to the questions ‘Did you finish it all?’ and ‘How do you feel?’ Once an entry was completed the consumption episode was logged in a food gallery. The food gallery consisted of a chronological slide show of the consumption episodes recorded so far during that day, and presented the meal photograph and all information recorded from the consumption experience questions. Users could navigate forwards and backwards through consumption episodes. Prior to deciding what and how much to eat for a meal during the day, users accessed this gallery to review everything they had eaten/drank so far that day. The application sent a notification reminding users to review the gallery shortly before their usual meal times. Users were able to programme what time they usually had breakfast, lunch and dinner on week days and weekends and were able to customise when the reminders were sent prior to that meal time. These components formed the main functions of the application and they were included in order to increase awareness of what was being consumed, enhance memory for eating episodes during the day and prompt users to think about previous eating episodes when making dietary decisions (see [20] for more detailed information).

An additional feature of the application that was added for this trial was an audio clip (2.5 min) that users could listen to whilst eating. The audio clip encouraged listeners to pay more attention to what they were eating, specifically by instructing users to pay attention to the smells, textures and tastes of the food whilst they were eating, as well as how full they felt. They were also encouraged to eat slowly, one mouthful at a time, and to periodically think about how much food was on their plate at the beginning and how much they had eaten (see Additional file 1 for audio clip transcript). The instructions were based on the audio clip instructions used in previous laboratory studies that have been found to enhance memory of recent eating episodes and reduce later snack intake [14, 15].

In order to motivate application usage, users could achieve in application ‘stars’ for reviewing the food gallery before a main meal, listening to the audio clip after taking a photograph of a main meal and completing a diary entry for a main meal (breakfast, lunch and dinner). An entry was considered complete when users took a photograph of the meal, completed the consumption experience questions after eating and the meal was logged in the gallery. Users were awarded a daily achievement badge if they obtained all available application stars during a single day. To further encourage regular use of the application, participants in the intervention group were told they would receive an additional £10 compensation (in addition to the £40 compensation all participants received for their time) for continued use of the application. Participants were told that continued use was recording most meals every day and listening to the audio clip a few times per week. In addition to the smartphone application participants also received a short paper based leaflet that explained the principles of attentive eating and other ways to eat attentively (e.g. avoiding eating whilst distracted). This resource can be found on the open science framework: 10.17605/osf.io/btzhw.

### Outcomes

#### Primary outcomes

Primary outcomes were body weight (kg) at 8 weeks and energy intake (kcal) at 4 and 8 weeks. Weight was measured with the Tanita BC-418 MA body composition analyser (Tanita Corporation, Tokyo, Japan). Two measures of energy intake were taken, self-reported 24 h energy intake and an objective laboratory measure of food intake in a bogus taste-test scenario [27]. Self-reported 24 h energy intake was measured using MyFood24, an online automated 24 h dietary assessment system developed and validated for use in the UK [28, 29]. Recommendations by the National Cancer Institute [30] suggest that the use of individual 24 h recalls pre and post intervention is a valid instrument to examine intervention effects on energy intake. The bogus taste-test is a laboratory standardised objective measure of food intake [27]. In the bogus taste-test participants were provided with 3 bowls of 50 g each of three biscuits (Maryland chocolate chip cookies ~ 249 kcal, Cadbury’s chocolate fingers ~ 240 kcal and McVities digestives ~ 241 kcal), broken up into small pieces to be comparable with previous laboratory studies. Participants were told that this was a taste perception task and given 10 min to rate the biscuits on 100-point visual analogue scales (anchors ‘not at all’ and ‘extremely’) on a number of features (e.g. crunchiness, flavoursome). Participants were also told that they could eat as many biscuits as they wished. Participants were asked not to eat for 1 h prior to the assessment sessions in order to standardise hunger. As some participants were attending the laboratory quite early (~ 8 am), asking participants not to eat for 1 h prior seemed reasonable. The biscuits were weighed afterwards in order to calculate the amount of biscuits consumed and converted to total kcals.

#### Secondary outcomes

Secondary outcomes were weight (kg) at 4 weeks and body fat percentage at 4 and 8 weeks. Both were measured with the Tanita BC-418 MA body composition analyser.

#### Additional outcomes

##### Ideal portion size at 8 weeks

One way eating attentively may reduce food intake is by altering beliefs about the satiating effects of food and in turn reducing ideal meal size. This was assessed using a computer-based visual portion size task, where participants were asked to indicate their ideal serving size for 18 meals; adapted version of Brunstrom and colleagues [31]. The average kcal content of the 18 meals was taken as the outcome measure. For more detailed information, see Additional file 1.

##### Self-reported trait eating behaviour at 8 weeks

Some studies have suggested that a more attentive approach to eating could reduce aspects of over-eating, binge eating symptoms, food cravings and increase awareness of internal signals of satiety when eating [32, 33]. Participants therefore completed the Three Factor Eating Questionnaire-21 [34] in order to assess aspects of over-eating (i.e. cognitive restraint, uncontrolled eating and emotional eating). Participants also completed the Binge Eating Scale [35], the Food Cravings Questionnaire [36] and the reliance on hunger and satiety cues sub-scale of the Intuitive Eating Scale [37].

##### Intervention efficacy beliefs at baseline and 8 weeks

To examine whether the intervention and control group participants differed in how effective they believed their intervention materials would be in reducing food intake and promoting weight loss during the baseline assessment (after randomisation) and at the 8 week visit, participants completed two questionnaire items measured using 100-point scales (anchors ‘not at all’ and ‘extremely’): ‘How confident are you that the materials and information provided to you during the study (e.g. paper based and mobile phone support) will help (helped) you eat less?’ and ‘How confident are you that the materials and information provided to you during the study (e.g. paper based and mobile phone support) will help (helped) you to lose weight?’

### Other measures and participant characteristics

Immediately prior to the taste-test, participants completed a 100-point visual analogue scale asking ‘how hungry do you feel right now?’ (anchors ‘not at all’ and ‘extremely’). Demographic information for each participant was collected: age, gender, ethnicity and education level achieved. Physical activity was assessed via the International Physical Activity Questionnaire (Booth, 2000). Semi-structured interviews were conducted with participants in the experimental group during the 8 week follow-up visit in order to understand participants’ experiences of the intervention (qualitative analyses using this data are to be reported elsewhere). We did not anticipate that adverse events related to the study would occur and so did not include a formal recording measure.

### Procedure

After being screened for eligibility via an online survey, participants attended the baseline visit where eligibility was re-confirmed in person (including measuring height and weight to verify BMI). Participants then provided consent to take part in the trial. Baseline measurements of body weight, height, body fat percentage, energy intake (both self-reported 24 h and objective laboratory bogus taste-test energy intake), trait eating behaviour and ideal portion size were then taken, and participants provided demographic information and completed the International Physical Activity Questionnaire [38]. Hunger was measured immediately before the taste-test on a 100-point visual analogue scale (anchors ‘not at all’ and ‘extremely’). Participants were then randomised to condition by the researcher. The researcher (a psychologist with a PhD) then explained the dietary advice booklet and the weekly text tips following a script. Participants in the intervention group were then told that they would also be using an attentive eating smartphone application. The same researcher introduced the concept of attentive eating and downloaded the application onto the participant’s mobile phone. The researcher then showed the participant how to use the different functions of the application, and how to personalise the timings of the in-app reminder notifications. Participants were then told that they would receive additional compensation for continued use of the application and were given the attentive eating take home leaflet. Participants in both groups were asked not to use any other structured (e.g. Weight Watchers) or smartphone based (e.g. My Fitness Pal) weight loss methods and to not take part in any other research during the trial. Participants then completed the intervention efficacy belief questions.

At the 4 week visit, body weight, body fat percentage and energy intake (both self-reported 24 h and objective laboratory bogus taste-test energy intake) were measured again. Hunger was again measured immediately before the taste-test. The researcher also asked participants about their use of the study materials. Participants in the intervention group were asked about their usage of the application and the researcher resolved any problems experienced with using the application in order to bolster use of the application for the remaining 4 weeks of the trial.

In the final 8 week visit, body weight, body fat percentage, energy intake (both self-reported 24 h and objective laboratory bogus taste-test energy intake), trait eating behaviour, ideal portion size and intervention efficacy beliefs were measured again. Hunger was measured immediately before the taste-test. Participants also completed the International Physical Activity Questionnaire. Semi-structured interviews were then conducted with participants in the intervention group.

### Statistical analyses

Statistical analyses were conducted in SPSS 24 [39]. Missing data for primary and secondary outcomes were imputed using multiple imputation implementing a multivariate imputation by chained equations logarithm. Twenty-two percent of participants had some missing data, and therefore 22 imputations were created. For the full list of variables included in the imputation models, see Additional file 1.

The effect of condition on primary and secondary outcomes was examined using regression analyses with 4 and 8 week measurements as the DVs, condition as the IV, controlling for baseline measurement of the DV and baseline BMI (as participants were stratified to condition based on BMI). Pre taste-test hunger was also controlled for in the analyses for objective laboratory measurement of bogus taste-test energy intake. To check the robustness of the multiple imputation results, the pooled results for primary outcomes were compared to two other approaches: last observation carried forward (LOCF) and complete cases only (CC). Results were considered significant if *p* < 0.05 for the main analyses.

For the additional outcomes analyses, missing data was imputed using the last observation carried forward method^1^ and the data was analysed using the same regression approach as for the primary and secondary outcomes. To account for multiple comparisons in our additional analyses, results were considered statistically significant if *p* < 0.01 for all additional outcome analyses. The two questions measuring intervention efficacy beliefs were correlated at baseline and at the 8 week assessment (both *r’s* = 0.8) and so the average of the two questions was calculated for each assessment session and used in the analysis to reduce multiplicity. In sensitivity analyses, we assessed the impact of protocol violations on the results for primary, secondary and additional outcomes by excluding data from participants who violated the protocol.

### Intervention adherence

Whether the proportion of trial days participants accessed the application predicted weight change at 8 weeks (intervention group and only those with follow-up data) was also examined, controlling for baseline weight using linear regression. In a per-protocol analysis we also compared participants who used the application as intended to the control group on primary and secondary outcomes using the same linear regression approach as for the main analyses. Participants who used the application as intended were defined as those who accessed the application on the majority of trial days and recorded 4 diary entries per day on at least half of the trial days. In addition, any participants who routinely completed entries immediately after taking the meal photograph (e.g. less than 1 min), with fewer than 0.5 gallery views per day and did not listen to the audio clip at all during the trial were excluded and not considered to be frequent users. There were 27 participants who used the application as intended. Results were considered statistically significant if *p* < 0.01, and analyses included only participants with follow-up data.

## Results

### Sample

**Fig. 1:**
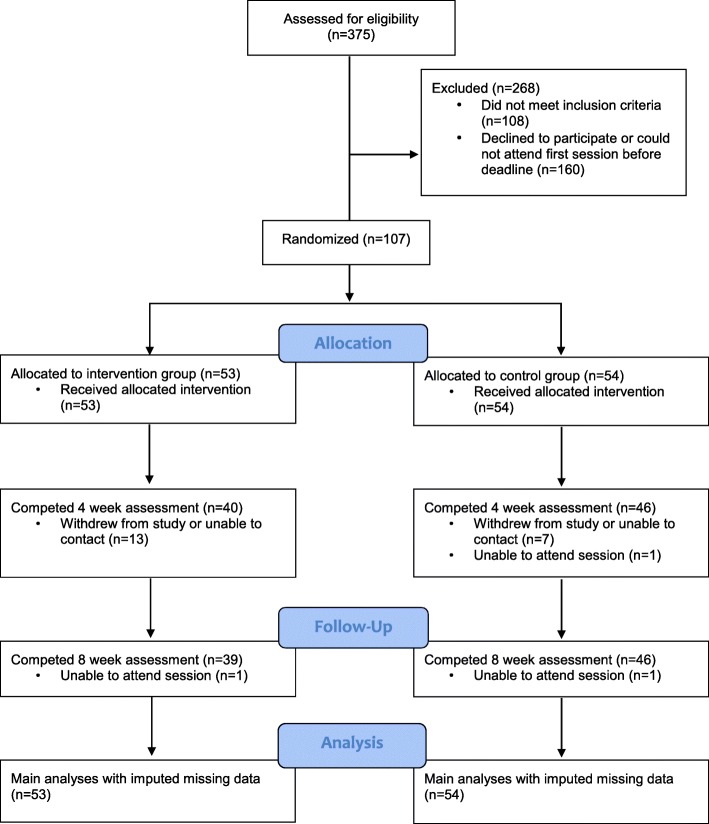
Flowchart of participants’ progress through the trial

Between September 2017 and February 2018, 107 participants were recruited and randomised to condition. Participants were on average 42 years old, mostly female, white and educated. See Table 1 for full sample characteristics. Eighty-six participants (80%) completed the 4 week assessment and 85 (79%) participants completed the 8 week assessment. There were slightly more drop outs in the intervention group than control group. All participants were maintained in the analyses (data imputed) for our main primary, secondary and additional outcomes. See Fig. 1 for the study flowchart.

**Table 1:** Baseline sample characteristics as a function of condition

	Intervention group mean (SD/%) *n* = 53	Control group mean (SD/%) *n* = 54
Age (y)	42.8 (10.5)	44.5 (10.7)
Gender (% female)	77.4	70.4
Ethnicity		
White	49 (92.5%)	51 (94.4%)
Mixed/Multiple	1 (1.9%)	1 (1.9%)
Asian/Asian British	3 (5.7%)	2 (3.7%)
Black/Black British	0 (0.0%)	0 (0.0%)
Other	0 (0.0%)	(0 (0.0%)
Education level^c^		
Entry level or equivalent	0 (0.0%)	4 (7.4%)
GCSE’s or equivalent	9 (17.0%)	8 (14.8%)
A/AS level or equivalent	12 (22.6%)	8 (14.8%)
Undergraduate degree or equivalent	20 (37.7%)	18 (33.3%)
Higher degree or equivalent	9 (17.0%)	15 (27.8%)
Other	3 (5.7%)	1 (1.9%)
BMI (kg/m^2^)	35.9 (6.8)	35.2 (6.2)
Weight at baseline (kg)	100.5 (20.4)	100.0 (17.6)
Body fat at baseline (%)	42.6 (8.0)	40.9 (8.2)
Self-reported energy intake at baseline (kcal)	2047.9 (696.6)	1944.0 (942.3)
Taste-test energy intake at baseline (kcal)	120.8 (105.0)	107.4 (101.8)
Ideal portion size (kcal)	455.7 (115.8)	459.1 (153.0)
Cognitive restraint^a^	2.3 (0.5)	2.3 (0.4)
Uncontrolled eating^a^	2.6 (0.5)	2.4 (0.5)
Emotional eating ^a^	2.6 (0.8)	2.5 (0.7)
Binge eating^a^	16.6 (7.6)	16.5 (7.5)
Reliance on hunger and satiety (intuitive eating)^a^	2.6 (0.7)	2.8 (0.8)
Food cravings^a^	72.9 (23.0)	71.3 (23.4)
MET minutes per week^b^	2473.2 (1793.0)	3431.9 (2683.8)

^a^Cognitive restraint, uncontrolled eating and emotional eating possible score range = 1–4; binge eating possible score range = 0–46; reliance on hunger and satiety (intuitive eating) possible score range = 1–5; food cravings possible score range = 21–126. Higher scores on all scales indicates greater endorsement

^b^MET minutes = metabolic equivalent minutes

^c^Percentages may not add up due to rounding

Groups appeared to be well balanced on baseline characteristics, except for physical activity MET (metabolic equivalent) minutes (see Table 1.). Including MET minutes as a covariate in the analyses for primary and secondary outcomes did not affect the results, therefore the results reported here are those without MET minutes as a covariate (as originally planned).

### Application usage

Participants who completed the 8 week assessment (*N* = 39) accessed the application on 74% (SD = 27%) of trial days, on average. The mean number of diary entries per day was 2.7 (SD = 1.6). The mean time taken to complete a diary entry (the time period between taking the photograph and answering the consumption experience questions) was 34.4 min (SD = 24.7 min), indicating that on average participants remembered to complete the entry within a reasonable time and thus were using the application as instructed. Participants listened to the audio clip on average 1.0 times a day (SD = 0.8), indicating good use of the audio clip.

### Primary and secondary outcomes

In the intervention group, mean weight change at 8 weeks (primary outcome) was − 1.2 kg (SD = 3.1), and − 1.1 kg (SD = 3.4) in the control group, with a mean difference of − 0.1 (95% CI = − 1.6, 1.3), which equated to a 1.1% decrease in body weight in both groups. There was no significant effect of trial condition on body weight, self-reported (24 h recall) or objective (laboratory measured) taste-test energy intake at 4 or 8 weeks. See Table 2. Use of LOCF and CC analyses did not affect the results for primary outcomes (not reported here).

**Table 2:** Pooled descriptive statistics and regression results for intervention effect on primary and secondary outcomes

	Intervention group mean (SD) (*n* = 53)	Control group mean (SD) (*n* = 54)	B (95% CI)	*p*
Primary outcomes				
Weight change 8 weeks (kg)	− 1.2 (3.1)	−1.1 (3.4)	− 0.1 (− 1.6, 1.3)	0.89
Self-reported energy intake change at 4 weeks (kcal)	− 396.0 (695.4)	− 326.9 (985.2)	3.7 (− 257.5, 264.9)	0.98
Self-reported energy intake change at 8 weeks (kcal)	− 216.5 (844.8)	− 223.9 (1017.2)	74.9 (− 273.4, 423.3)	0.67
Taste-test energy intake change at 4 weeks (kcal)	3.0 (75.8)	21.9 (87.8)	−23.7 (− 56.2, 8.9)	0.15
Taste-test energy intake change at 8 weeks (kcal)	41.4 (86.7)	17.7 (95.4)	25.1 (− 12.1, 62.3)	0.19
Secondary outcomes				
Weight change at 4 weeks (kg)	− 0.7 (2.1)	−0.7 (2.2)	− 0.1 (− 1.0, 0.8)	0.88
Body fat change at 4 weeks (%)	0.1 (2.3)	−0.5 (2.0)	0.6 (− 0.4, 1.5)	0.24
Body fat change at 8 weeks (%)	−0.4 (1.8)	− 0.5 (2.0)	0.1 (− 0.7, 0.9)	0.81

### Additional outcomes

There was no effect of trial condition on ideal portion size, cognitive restraint, uncontrolled eating, emotional eating, reliance on hunger and satiety (intuitive eating), binge eating and food cravings at 8 weeks. See Table S1 in Additional file 1. There was also no evidence that the intervention significantly affected efficacy beliefs at baseline, B = 7.1, 95% CI -0.9 to 15.0, *p* = 0.08, and at 8 weeks, B = 9.2, 95% CI -1.6 to 19.9, *p* = 0.09.

### Sensitivity analyses

Excluding participants due to protocol violations (*n* = 4: 1 likely measurement error for weight, 2 tried alternative weight loss methods during the trial, 1 did not receive all of the text tips) had no effect on the results for primary, secondary and additional outcomes.

### Intervention adherence analyses

The proportion of trial days that intervention group participants used the application did not significantly predict weight at 8 weeks, B = − 1.8, 95% CI -4.3 to 0.7, *p* = 0.15. The per protocol analyses (*n* = 27 in intervention group vs. *n* = 45 in control group) revealed no significant effects of trial condition on weight, body fat percentage, self-reported 24 h or objectively measured taste-test energy intake. See Table 3.

**Table 3:** Descriptive statistics and regression results for intervention effect on primary and secondary outcomes in the per protocol analyses

	Intervention group mean (SD) (*n* = 27)	Control group mean (SD) (*n* = 45)	B (95% CI)	Beta	*p*
Baseline					
Weight at baseline (kg)	95.2 (22.2)	100.4 (17.8)			
Body fat at baseline (%)	41.5 (9.0)	40.3 (8.5)			
Self-reported energy intake at baseline (kcal)	1968.2 (646.6)	2042.6 (981.5)			
Taste-test energy intake at baseline (kcal)	121.3 (97.5)	106.4 (93.8)			
Primary outcomes					
Weight change at 8 weeks (kg)	− 1.2 (2.2)	− 1.1 (3.0)	− 0.3 (− 1.6, 1.1)	−0.01	0.71
Self-reported energy intake change at 4 weeks (kcal)	− 322.2 (513.6)	− 406.8 (989.8)	27.3 (− 220.4, 275.0)	0.03	0.83
Self-reported energy intake change at 8 weeks (kcal)	− 220.9 (681.7)	− 300.0 (989.8)	25.7 (− 297.8, 349.2)	0.02	0.87
Taste-test energy intake change at 4 weeks (kcal)	− 1.3 (75.5)	14.4 (85.6)	−19.5 (−57.1, 18.2)	−0.1	0.31
Taste-test energy intake change at 8 weeks (kcal)	38.6 (71.8)	12.3 (90.3)	29.6 (−8.6, 67.7)	0.1	0.13
Secondary outcomes					
Weight change at 4 weeks (kg)	−0.8 (1.2)	−0.7 (2.0)	− 0.2 (−1.0, 0.7)	−0.004	0.69
Body fat change at 4 weeks (%)	0.2 (2.2)	−0.5 (1.6)	0.7 (− 0.2, 1.6)	0.04	0.12
Body fat change at 8 weeks (%)	− 0.2 (1.3)	−0.4 (1.8)	0.1 (− 0.7, 0.9)	0.01	0.75

## Discussion

In the first randomised control trial testing the efficacy of an attentive eating smartphone application, we found no effect of the intervention on energy intake or body weight in adults seeking to lose weight. Sensitivity analyses for loss to follow-up, protocol violations and per protocol analyses did not change the results. There was also no evidence that the intervention affected other aspects of eating behaviour.

One possible explanation for finding that the attentive eating smartphone application did not produce greater reductions in weight or eating habits than the control group is that participants did not use the application enough to promote these changes. However, application usage data indicates that participants tended to use the mobile phone application frequently. Furthermore, although smaller in sample size, analyses comparing participants who used the application as intended vs. the control group did not show any effects of intervention on weight loss and energy intake. The proportion of trial days participants accessed the application also did not predict weight loss. Poor usage of the application appears unlikely to explain the lack of effect. Across both the intervention group and control group participants lost a modest amount of weight by 8 weeks (1.1–1.2 kg) and self-reported 24 h energy intake was lower at 4 and 8 weeks compared to baseline in both groups. This amount of weight loss is similar to that observed among people losing weight without behavioural support [40]. These modest weight losses imply, however, that the lack of effect is not due to ceiling effects i.e. users adhering to such strict dietary regimens that the application had no prospect of effect.

Short-term laboratory studies suggest encouraging an attentive eating style reduces food intake in participant with healthy weight and overweight [14–16]. However, other recent laboratory research did not replicate these effects or demonstrate any effects of focused attention on memory for recent food intake [17]. Given that the present study was the first to examine the applied relevance of attentive eating outside of the laboratory it is difficult to conclude whether the impact attentive eating has on food intake has been overestimated in laboratory studies or whether other aspects of the present intervention study design explain the lack of effect of attentive eating on energy intake and weight loss. For example, laboratory studies do not tell participants about the value of and aims of eating attentively, but this might be expected to increase not decrease the effectiveness of an intervention.

Understanding why the attentive eating application did not produce significant weight loss may be informative. One possibility is that increased attention to food during active weight loss does not promote further reduced food intake because it reminds individuals that they are eating less than usual. Another possibility is that the application did not have the intended psychological impact. Although we were able to record participant adherence to the intervention we were unable to include a direct measurement of the extent to which the smartphone application increased attention to food during eating and improved memory of food eaten.

### Strengths and limitations

Given that this was the first proof-of-concept trial of the effect of attentive eating, energy intake and weight loss were examined at only 4 and 8 week follow up. Although this approach is consistent with other proof-of-concept trials examining the effect of psychologically informed weight loss interventions [24], it prevents us from making conclusions about the longer term effectiveness of attentive eating. However, we know of no intervention that leads to long-term weight loss that does not also lead to short-term weight loss and thus we can be reasonably confident that this intervention is unlikely to be effective in the long-term. A limitation of the current study is that only 25% of the study sample was male, which is not representative of the UK population [41], but is similar to other weight loss trials [42]. A strength of the present study was the use of an active control group [22, 23] and that our control and intervention group were rated similarly by participants in terms of expected efficacy, as a failure to account for expectancy effects when examining efficacy of behavioural interventions can lead to incorrect conclusions about intervention efficacy [43]. A further strength of the current study was the use of both self-reported and objective measures of food intake, as these measures used in isolation both have their weaknesses [44, 45]. The consistent non-significant effect of the intervention on both measures of energy intake supports the robustness of the findings, regardless of the limitations of the individual methods. The robustness of the trial findings is also supported by the consistency of the results across the several methods used to handle missing data and the sensitivity analyses. Further, the dropout rate in the present trial (21%) is similar to that observed in other weight loss trials [46, 47]. We were not able to measure whether the attentive eating application improved memory for recent eating during the trial, so although we can conclude the attentive eating application was ineffective in promoting weight loss, it is less clear whether it is a useful tool by which to study the effects of memory for recent eating outside of the laboratory. Future work would benefit from more directly measuring the effect of the application on attention paid to food and memory for recent eating. Further, it is likely that participants did not report all meals and drinks consumed, however adherence to the application was reasonable. Whilst there was a reasonable range of intake in the bogus taste-test, intake was less than observed in laboratory studies that are not weight loss trials [15], suggesting that there could have been a floor effect on food intake in the bogus taste-test. In addition, we did not measure participants’ history of weight loss attempts or use of dietary mobile phone applications which may be useful to measure in future research for descriptive purposes.

## Conclusions

A smartphone based attentive eating intervention and standard dietary advice had no effect on energy intake or weight loss at 4 or 8 week follow-up compared to standard dietary advice alone.

## Additional file

Additional file 1: For ‘A smartphone based attentive eating intervention for energy intake and weight loss: results from a randomised controlled trial’. This file contains several pieces of information supplementary to the main manuscript, including further information on methodology and statistical results. (DOCX 22 kb)

## Abbreviations

BMI: Body mass index
CC: Complete case
DV: Dependent variable
IV: Independent variable
LOCF: Last observation carried forward
MET: Metabolic equivalent

## Footnotes

1 In the pre-registered protocol we planned to use multiple imputation to impute missing data for additional outcomes, however, values for additional outcome variables could not be obtained within a reasonable number of case and parameter draws, and so last observation carried forward was used instead.
