# Supplementary material for: A smartphone based attentive eating intervention for energy intake and weight loss: results from a randomised controlled trial
Source: BMC Public Health. 2019 May 21;19:611. doi: 10.1186/s12889-019-6923-x (PMC6528285; doi:10.1186/s12889-019-6923-x)
Supplement: Supplementary file 1 — For ‘A smartphone based attentive eating intervention for energy intake and weight loss: results from a randomised controlled trial’. This file contains several pieces of information supplementary to the main manuscript, including further information on methodology and statistical results. (DOCX 22 kb) [file 12889_2019_6923_MOESM1_ESM.docx]

**Additional file 1 for ‘A smartphone based attentive eating intervention for energy intake and weight loss: results from a randomised controlled trial.’**

**Focused attention audio clip transcript**

We would like you to follow some guidelines while you are eating.

Before you begin eating your food, think back to what you have already eaten today so far. How many meals and snacks have you eaten today?

Take a moment to look at the food in front of you.

Look at the shape, colour, markings, contours, and textures of the food. Also pay attention to the smell of the food, think about what the smell is like- is it similar to any other smells you know? And what different smells can you detect?

Now you can start eating your food. After putting food in your mouth, return your hands to a resting position before you begin chewing. Your hands should be empty whilst you are eating. Eat the food one mouthful at a time, slowly and carefully, and remember not to put any more in your mouth until you have completely finished what you are chewing.

Continue eating whilst you think about the following things.

Notice the initial flavour of the food- what does it taste like? And what different flavours can you detect? Notice any differences in taste in different parts of your mouth. Can you detect sweetness, sourness or saltiness?

Notice the textures of the food and how they change as you chew.

Pay attention to how the flavour changes as you chew.

Become aware of the speed of your chewing.

Notice the position and movement of your tongue, jaw, and teeth whilst you are chewing and swallowing.

After you swallow each bite, please pay attention to the aftertaste that develops- are the flavours you sense during the aftertaste the same as the flavours during chewing? And how does the aftertaste change after you have swallowed?

Continue to think about these things while you eat more of your food. Remember to think about flavour, taste, textures, sounds, speeds and movements.

After you have eaten some of the food, pause to remember how much food there was at the beginning. Notice how much of the food you have eaten and how much you have left.

And when you have finished the meal take a little time to think about how full you feel.

**Ideal portion size task**

In this task participants were shown portion size photographs of a meal on the screen, and were asked to use buttons on the keyboard to adjust the portion size. Based on their responses for the 18 meals, participants were assigned a value (in kcals) that represents the average energy content of their ideal portion size. Participants were also asked to state whether they had eaten the 18 meal foods before to check familiarity with the foods^[[1]](#endnote-1)^. Meal items that were familiar to less than 50% of trial participants was excluded from the ideal portion size task^[[2]](#endnote-2)^. Only 28% of trial participants reported that they had eaten the grilled fish meal before, and only 49.5% of participants had eaten the vegetable biryani before. These two dishes were therefore excluded, leaving 16 meal items in the ideal portion size task.

**Statistical analyses**

Variables included in the imputation models were: trial arm allocation, age, gender, ethnicity (white vs. not), education level at baseline; body weight, body fat percentage, energy intake (self-reported 24 hour energy intake and objective laboratory measured energy intake) and pre taste-test hunger at baseline, 4 weeks and 8 weeks; ideal portion size, cognitive restraint, uncontrolled eating, binge eating symptoms, food cravings, intuitive eating (reliance on hunger and satiety cues), physical activity (total MET minutes) and intervention efficacy beliefs at baseline and 8 weeks.

**Results**

**Additional outcomes**

**Table S1.** Change in additional outcomes from baseline to 8 weeks.

|  | App group mean (SD)  N = 53 | Control group mean (SD)  N = 54 | B (95% CI) | Beta | *p* |
| --- | --- | --- | --- | --- | --- |
| Ideal portion size (kcal) | -39.9  (94.1) | -44.1^a^  (121.8) | 1. 1  (-35.3, 37.6) | 0.005 | 0.95 |
| Cognitive restraint^b^ | 0.2  (0.4) | 0.3  (0.5) | -0.1  (-0.2, 0.1) | -0.1 | 0.54 |
| Uncontrolled eating^b^ | -0.1  (0.4) | -0.2  (0.4) | 0.1  (-0.1, 0.2) | 0.1 | 0.37 |
| Emotional eating^b^ | -0.1  (0.5) | -0.1  (0.4) | -0.035  (-0.2, 0.1) | -0.023 | 0.67 |
| Binge eating^b^ | -1.3  (5.7) | -2.3  (5.8) | 0.9  (-1.1, 3.0) | 0.1 | 0.38 |
| Reliance on hunger and satiety (intuitive eating)^b^ | 0.3  (0.7) | 0.1  (0.6) | 0.2  (-0.02, 0.4) | 0.1 | 0.08 |
| Food cravings^b^ | -4.3  (13.1) | -4.8  (17.1) | 0.8  (-4.9, 6.4) | 0.016 | 0.79 |

^a^Data missing for one participant.

^b^Cognitive restraint, uncontrolled eating and emotional eating possible score range = 1-4; binge eating possible score range = 0-46; reliance on hunger and satiety (intuitive eating) possible score range = 1-5; food cravings possible score range = 21-126. Higher scores on all scales indicates greater endorsement.

1. Checking familiarity with the meal food items was not described in the pre-registered protocol in error. [↑](#endnote-ref-1)
2. Excluding meal items familiar to less than 50% of participants were not stated in the pre-registered analysis plan in error. However, this is typically done for this task (see Whitelock, Higgs, Brunstrom, Halford, & Robinson, 2018). [↑](#endnote-ref-2)
